# Barriers and facilitators influencing the implementation of the occupational health intervention ‘Dynamic Work’: a qualitative study

**DOI:** 10.1186/s12889-022-13230-9

**Published:** 2022-05-11

**Authors:** Victoria J. E. Z. Mastenbroek, Judith G. M. Jelsma, Hidde P. van der Ploeg, Dominique P. M. Stijnman, Maaike A. Huysmans, Allard J. van der Beek, Femke van Nassau

**Affiliations:** Department of Public and Occupational Health, Amsterdam UMC, Vrije Universiteit Amsterdam, VU University Medical Center, Amsterdam Public Health Research Institute, Van der Boechorststraat 7, Amsterdam, 1081 BT The Netherlands

**Keywords:** Sedentary behavior, Occupational health, Intervention, Sitting time, Implementation, Facilitators, Barriers, Office workers, Qualitative study

## Abstract

**Background:**

Sedentary behavior is associated with an increased risk of morbidity and mortality. To reduce occupational sitting time of office workers, the multi-component intervention ‘Dynamic Work’ was implemented in a Dutch insurance company. Although the results showed no significant reductions in sitting time, associations were found between higher levels of implementation and reductions in sitting time. Building upon these findings, this qualitative study aimed to identify barriers and facilitators from an organizational perspective for the implementation of Dynamic Work. In addition, we explored differences in barriers and facilitators between departments with a low, middle and high level of implementation.

**Methods:**

In total, eighteen semi-structured interviews were conducted with two Dynamic Work coordinators, three occupational physiotherapists who delivered the intervention, and thirteen department managers. All participants were purposively sampled. The data was coded in Atlas.ti and a thematic analysis was performed guided by The Integrated Checklist of Determinants (TICD).

**Results:**

Implementation factors were related to the organization; working culture and financial support facilitated implementation. Factors related to the implementing department mainly hindered implementation, i.e. lack of information at start of the project, late delivery of Dynamic Work equipment, large group sizes, employee’s workload and work tasks, and an ongoing reorganization. The facilitating role of managers was experienced as both enabling and hindering. The pre-existing familiarity of the occupational physiotherapists with the departments and alignment amongst the three implementers facilitated implementation. Yet, the non-obligatory nature of the intervention as well as limited availability and technical problems of equipment did not support implementation.

**Conclusions:**

Various barriers and facilitators influenced the implementation of the Dynamic Work intervention, where the key role of the department manager, late delivery of dynamic work equipment and groups sizes varied between low and high implementing departments. These results can contribute to developing and improving implementation strategies in order to increase the effectiveness of future occupational health interventions.

**Trial registration:**

The study protocol was registered on April 14, 2017 in the ClinicalTrials.gov Protocol Registration and Results System under registration number NCT03115645.

**Supplementary Information:**

The online version contains supplementary material available at 10.1186/s12889-022-13230-9.

## Background

Sedentary behavior has been found to be a health risk [1]. High levels of sedentary behavior are consistently associated with an increased risk of cardiovascular disease, type II diabetes, hypertension, depression, musculoskeletal problems, and premature death [2]. In 2013, over 18% of European adults sat more than 7.5 h per day, with office workers spending more than 60% of their workdays sitting at work [1, 2]. As a result, sedentary behavior has been estimated to cause over 1 million deaths and to cost up to €80.4 billion per year in Europe in 2012 [3, 4]. Evidence, however, indicates that short bouts of standing and light activity interrupting long sitting periods can reduce cardiometabolic risk markers, such as insulin, triglycerides and high blood pressure [5]. Hence, occupational sitting time can serve as a primary target for interventions to reduce sedentary behavior [6].

Research has shown that multi-component interventions are effective at reducing sitting time when targeting a combination of physical workplace changes, policy changes, providing information and counselling [6, 7]. The implementation of multi-component health interventions are, however, complex processes, as these are often extensive interventions involving many different stakeholders and workplace settings vary in sector, organizational structure and culture [2, 8, 9].

Previous research has already identified over 50 barriers and facilitators that potentially influence the implementation of worksite health promotion programs [10]. For example, strong management support was most frequently mentioned facilitator, whereas no fit with the organizational culture and a lack of resources were most frequently mentioned barriers for implementation [10]. In another systematic review, intrinsic factors related to participants (e.g. commitment), as well as extrinsic factors related to the organization (e.g. flexibility of the intervention, nature of work tasks, and managerial support) were found to affect the uptake of workplace health promotion interventions [3].

In 2017, the multi-component intervention ‘Dynamic Work’ was implemented at a Dutch insurance company aimed to reduce sitting time among office workers [11]. The intervention was delivered by internally employed occupational physiotherapists and consisted of an individual, organizational and environmental component. Contrary to expectations, no statistically significant differences were found in total sitting time per day between the intervention and control group at 8-month follow-up. However, moderate associations were found between reductions in sitting time and higher levels of implementation in participating departments (-8 min/day) [9]. Yet, it is unknown what barriers and facilitators influenced intervention implementation, and whether these barriers and facilitators differ across workplaces with varying degrees of implementation. Therefore, the objective of this study was to identify barriers and facilitators from an organizational perspective influencing implementation of Dynamic Work, and secondly, to explore differences in barriers and facilitators between departments with a low, middle and high level of implementation.

## Methods

### Study design and setting

In this qualitative study, semi-structured interviews were conducted as part of the larger Dynamic Work study. The intervention was implemented at eight departments of a large Dutch insurance company, which varied in size from seven to 55 employees. Employees’ work tasks consisted of a combination of desk work, attending meetings and visiting customers. Employees usually worked from home for 1–2 days per week.

### Dynamic Work intervention

Dynamic Work is a multi-component intervention aimed to decrease total sitting time among office workers [7]. More information about the intervention can be found in the study protocol [11]. The intervention was designed by two Dynamic Work coordinators from the company’s occupational health department, together with three internal occupational physiotherapists and an external research partner. The intervention was delivered by the occupational physiotherapists, who were each responsible for implementing the intervention in certain departments. Managers from all participating departments had an initial face-to-face meeting with the occupational physiotherapist, in which their potential motivating role and logistics were discussed. Motivation of the managers consisted of informing them about the program, defining a time schedule for the sessions and discussing the manager’s role in supporting their employees in a less sedentary work life. The intervention furthermore contained components at the environmental (e.g. sit-stand desks, cycling workstations and office balls), organizational (e.g. face-to-face meeting between an occupational physiotherapist and a department manager) and individual level (e.g. self-help program booklets with an action plan and advice for behavior change, activity monitor called the Activator for real life feedback on sitting time and steps).

Two group sessions (30 min each) were scheduled one month apart from one another and consequently four onsite meetings per department were delivered by the occupational physiotherapists). During the group sessions, health risks associated with prolonged sitting and correct usage of the environmental component were explained and personal experiences with the intervention components were exchanged. During the onsite meetings, the occupational physiotherapists were available to answer questions from participants regarding the use of the intervention components. A randomized controlled trial was performed to evaluate the effectiveness of Dynamic Work [7]. In addition, a process evaluation was conducted to provide insight into the working and failure mechanisms of Dynamic Work [9].

### Data collection

A purposive sampling technique was used to recruit respondents. To achieve data saturation, all relevant stakeholder groups, which were involved in the implementation process, were included. Study participants consisted of two Dynamic Work coordinators, who coordinated implementation, three occupational physiotherapists, who delivered the intervention, and thirteen department managers representing all departments. After receiving informed consent, one-on-one semi-structured interviews were conducted in May 2018 using three different topic guides tailored for each type of respondent. The topic guides were developed building upon The Integrated Checklist of Determinants of practice (TICD) domains [12], and tailored to the specific stakeholder, in order to obtain in-depth information from their involvement in the implementation process. The topic guides can be found in the appendices [see Additional files 1, 2 and 3]. The interviews took place both on location and over the phone. At the beginning of each interview, respondents were asked for their demographic data (e.g. age, work experience, role in Dynamic Work), followed by the following domains: recruitment of departments, recruitment of occupational physiotherapists, recruitment of participants, program delivery, views and experiences with the intervention, barriers and facilitators for implementation, and sustainability. Department managers also provided a short description of their department. The interviews were conducted by two senior researchers (J.J. and F.vN.) with previous experience in qualitative research. J.J. coordinated the Dynamic Work project and interviewed only department managers. F.vN. had no previous role in the implementation of Dynamic Work and therefore interviewed the Dynamic Work coordinators, occupational physiotherapists, and department managers. All interviews were recorded, transcribed verbatim and anonymized. The interviewers wrote field notes after each interview to generate contextual knowledge.

### Data analysis

A thematic analysis was performed to identify barriers and facilitators [13]. A codebook was therefore developed by F.vN., M.H., J.J. and V.M. For this purpose, the transcripts were first read to gain familiarity with the data. After that, the transcripts were open coded. The open codes were subsequently compared in a group meeting and clustered in overarching codes, which were then categorized into one of the seven domains from TICD [12]. The names of the original TICD domains were, however, adjusted to better reflect factors relevant to Dynamic Work, as shown in an additional file [see Additional file 4]. With the final codebook that emerged from this procedure [see Additional file 4], F.vN. and V.M. independently coded three interviews and discussed any coding discrepancies until consensus was reached. V.M. subsequently coded the remaining transcripts. Coding was performed using Atlas.ti (version 8.4.24.0). Finally, overarching themes and subthemes were identified by analyzing the existing codes and corresponding quotations.

As part of the process evaluation, an implementation index score was constructed for each department [9]. This score was calculated based on nineteen theory-derived process-items reported by intervention participants across four domains. These domains were: 1) dose (with items related to meeting attendance), 2) adherence (with items related to usage of Dynamic Work components), 3) quality of delivery (with items related to need for support given by occupational physiotherapists), and 4) participant responsiveness (with items related to use of behavioral change techniques by participants). We then aggregated an implementation index score per department. Based on these implementation index scores, departments were divided into low, middle and high implementing departments to enable comparisons between barriers and facilitators on departments with a low, middle and high level of implementation.

### Ethics

The Medical Ethics Review Committee of the VU University Medical Center Amsterdam (2016.533) approved the study and all participants provided written informed consent.

## Results

In total, 18 interviews were conducted with occupational physiotherapists (*n* = 3), Dynamic Work coordinators (*n* = 2), and managers (*n* = 13) (see Table 1). Interviews with occupational physiotherapists lasted longer than those with coordinators and managers.

**Table 1:** Participant characteristics

	**Occupational physiotherapists (*n* = 3)**	**Dynamic Work coordinators (*n* = 2)**	**Managers (*n* = 13)**
**Gender**			
Men: n (%)	3 (100)	1 (50)	7 (54)
**Age (years)**			
Mean (SD); range	40.7 (14.7); 27–61	44.5 (3.5); 41–48	41.0 (6.7); 28–51
**Work experience (years)**			
Mean (SD); range	9.7 (6.5); 3–18	13.0 (4.0); 9–17	11.0 (4.5); 5–17
**Interview duration (minutes)**			
Mean (SD); range	82.3 (6.2); 77–91	60.5 (0.5); 60–61	27.9 (5.1); 21–40
**Department**			
Low implementing department (*n* = 3)	n/a	n/a	5
Middle implementing department (*n* = 3)			4
High implementing department (*n* = 2)			4

*N* Number*, SD Standard deviation*

The department implementation index scores varied from 8.5 (± SD 2.1) to 12.1 (± SD 2.3). Two departments with a score below 9 were considered ‘low implementers’, three departments with a score between 9 and 10 were considered ‘medium implementers’, and three departments with a score above 10 were considered ‘high implementers’.

**Fig. 1:**
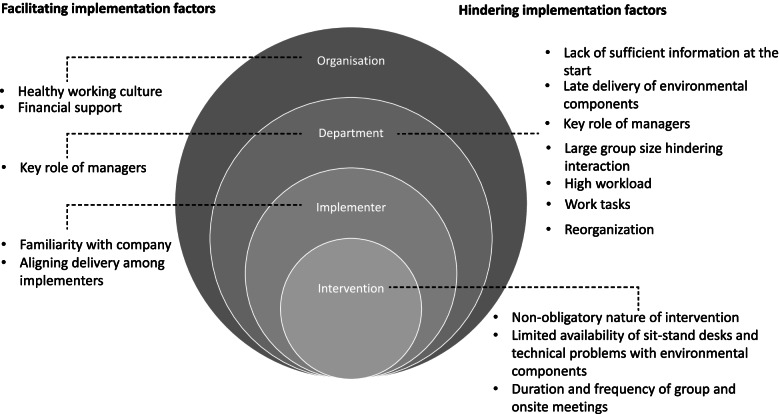
Overview themes

The analysis of implementation barriers and facilitators across all seven domains of the TICD checklist is provided in Appendix 4. Figure 1 provides an overview of themes across the level of the organization, department, implementer (occupational physiotherapists) and intervention.

### Factors related to the organization

This theme captured factors on the organizational level: healthy working culture and financial support.

#### Healthy working culture

It was expressed that the insurance company constantly invests in the health of their employees, resulting in a perceived healthy working culture and mindset of employees. This facilitated a genuine interest in participants' own health and stimulated healthy behaviors while at work by making use of available intervention components.

“*[The Dynamic Work coordinator] had this strong vision that dynamic working is visible. What we’re doing for healthy working is all behind closed doors. The psychologist is here too, talking to a patient (…) Nobody sees that, and they shouldn’t, but dynamic work is very visible (….) and employees of [the insurance company] can see that and that’s how it worked.*”—Dynamic Work coordinator 2.

#### Financial support

The board of directors financially supported the health of their employees, which facilitated roll out of the intervention (i.e. participation was free of charge for employees) and intervention adaptation possibilities (i.e. financing additional meetings when needed).

“*Then [occupational physiotherapist] said: ‘yes, I do not really feel like I have reached everyone’ (…). ‘I’d better visit them again [Dynamic Work coordinator], is that okay with you?’. Because I have to pay for it. (…). And then I said: ‘Well you know, I find it more important that people become active, so you just decide how often you think is necessary to visit in order to reach everyone.*”—Dynamic Work coordinator 1.

### Factors related to the department

This theme captured factors on the department level: lack of sufficient information at the start, late delivery of environmental components, key role of managers, high workload hindering participation, work tasks hindering participation and reorganization hindering participation.

#### Lack of sufficient information at the start

Dynamic Work coordinators recruited departments for the intervention. They mainly relied on existing relationships with managers. Yet, occupational physiotherapists expressed that they felt that some department managers were not well informed by the Dynamic Work coordinators prior to the start, which appeared to be a large barrier for implementation.

“*What also played a role is that we felt like managers didn’t realize the impact this would have. (…). I think [the Dynamic Work coordinator] also didn’t realize that very well and that as a result, [the Dynamic Work coordinator’s] information to the managers was not, uhm very clear. (…) And then we would get there with our group session and Activators, et cetera (….) and you see the people there being hesitant or not getting the point and that stings a little bit sometimes.*”—Occupational physiotherapist 3.

This hindered both the amount of time managers devoted to the intervention and the extent to which they stimulated their employees to participate. In some departments, it was decided from upper management that departments would participate, which caused managers to be even less supportive of the intervention.

#### Late delivery of environmental components causing suboptimal timing between intervention components

In many low and middle implementing departments, the facility department was later than scheduled (up to two months delay) with delivering the environmental components. This resulted in multiple reschedules of group sessions and in a suboptimal timing between intervention components overall. According to multiple respondents, this led to a decreased motivation of employees to use the facilities.

“*In some cases, the first group session was already scheduled and it was promised that the furniture would be there in time, but it was not, so the session was given first before the equipment arrived a month later. Well, that was actually a bit of a waste of the first workshop (…). Or the furniture was already there for two months and the workshop was delayed (…) because the manager cancelled it twice.*”—Dynamic Work coordinator 1.

#### Large group size hindering interaction

In one low implementing department, the kick-off meeting was held with a much larger group than the occupational physiotherapists had originally planned**.** This limited the amount of interaction and hindered the impact of that meeting.

“*The first plenary session, which is of course very educational, and in that sense was more about sending information, we made the concession to give it for the whole department. Yes, that is really 70, 80 people (…). I noticed that you miss impact there, because if you give it in a team meeting with 12 people for half an hour and people talk about it, the subject resonates much more within such a [small] group*.”—Occupational physiotherapist 1.

Contrary, the group meetings of high implementing departments took place in smaller groups and were characterized by a lot of interaction among participants.

#### Key role of managers throughout implementation

Managers played a key role in the initial decision for department participation and actual implementation of the intervention, by serving as a role model to their employees, and openly talk about the intervention. Managers of departments with a high and middle level of implementation mentioned that they stimulated their employees regularly to participate, for instance by addressing the topic in team meetings and personal conversations.

“*You see such a [manager] there. (…) What you see there is that she really embraces it. She is an example as she is practicing it herself; she is standing behind a desk herself.*”—Occupational physiotherapist 1.

Although managers of departments with a low level of implementation mentioned they think managers serve as a role model to their employees, they mentioned that they did not talk to employees about the intervention and did not engage with the implementation in any way.

Additionally, the occupational physiotherapists mentioned that it was difficult to plan an initial meeting with managers of departments with a low level of implementation, and due to other priorities they more often started negotiating about intervention components that cost time.

“*The managers have to be on board for the planning (…) you notice that people find it difficult to make time for it, because they all have targets and things they have to achieve. (…). And if the manager is not fully on board, it becomes very difficult.*”—Occupational physiotherapist 2.

#### High workload hindering participation of employees

Occupational physiotherapists and managers from all departments mentioned that the high workload hindered many employees in fully committing to the intervention. Work-related tasks were often prioritized by managers over participation in the Dynamic Work intervention.

“*R: I believe it’s going better now with [the insurance company], but last year and the year before that, results were very bad. Yes, then it is hard to get the attention for this.*

*I: Because managers are not held accountable for these projects?*

*R: Yes, not at all.*

*I: But they are on their core targets (…) so they have to balance that.*

*R: These are of course all side issues.*”—Dynamic Work coordinator 2.

#### Work tasks hindering participation of employees

In many departments, employees regularly worked from home or attended meetings at external locations. As a result, they were often absent from group sessions and onsite meetings, and had fewer opportunities to use the dynamic facilities. The nature of some employees’ work also hindered them in using the dynamic facilities, because they needed a specific workplace due to double computer screens or specific trays.

#### Reorganization hindering participation of employees

At the time of implementation, the insurance company was in the middle of a large reorganization. On the one hand, employees were affected by the uncertainty about continuation of their job. One department (middle level of implementation) included in this study was highly affected as half of the employees in that department lost their job. On the other hand, most departments were housed in fewer locations, which resulted in a situation that more employees needed to share dynamic facilities.

### Factors related to the implementer

This theme captured factors on the implementer level: familiarity with company, aligning delivery among implementers.

#### Familiarity with company

Occupational physiotherapists could, due to being employed prior to this project, easily visit departments as they were already present on the locations. Existing relationships with managers facilitated implementation. Furthermore, this intervention fitted well with the occupational physiotherapists’ regular work tasks, such as regularly treating employees for physical complaints. This facilitated responsiveness in employees, because employees were likely to trust the occupational physiotherapists due to their expertise.

“*I think it is truly part of our work (…) getting people active and educating them. We work with physical complaints of course, so physical activity is also very important for that. And I think it is very good to contribute to that.*”—Occupational physiotherapist 2.

#### Aligning delivery among implementers

Occasional meetings between the occupational physiotherapists facilitated implementation. Those meetings allowed for aligning delivery and learning from each other’s experiences. All occupational physiotherapists had diverse delivery styles, different backgrounds, experiences and expertise, which further strengthened implementation.

“*I: You are a team, but from what I understood, everyone has his own locations and does his own thing a little bit.*

*R: Yes, we are quite independent, but I think we also strengthen each other (…) with our expertise (…). We all kind of have a bit different background, mine is sport-related, [occupational physiotherapist 1] psychosomatic [occupational physiotherapist 3] and also manual therapy, ergonomics (…). So very different perspectives.*”—Occupational physiotherapist 2.

### Factors related to the intervention

This theme captured factors on the intervention level: non-obligatory nature of intervention, limited availability of sit-stand desks and technical problems with environmental components, and duration and frequency of group and onsite meetings.

#### Non-obligatory nature of intervention

One manager and one Dynamic Work coordinator found the intervention to be too much without obligation, which limited the intervention’s potential impact.

*“I do think when it comes to mindset and awareness, it is a good initiative and it is important that it happened within the team. I do think it has been without too much obligation. With regard to both its implementation and what we got out of it.”*—Manager 11.

#### Limited availability of sit-stand desks and technical problems with environmental components

Limited availability of sit-stand desks resulted in sit-stand desks often being occupied for prolonged periods of time by the same employees, which prevented others from using them. Additionally, various technical problems and already owning advanced smartwatches hindered use of the Activator activity monitor. Technical problems also arose for some cycling workstations (i.e. the pedals broke) and the cycling workstations and the office balls were considered unpractical for tall people.

#### Duration and frequency of group and onsite meetings

The occupational physiotherapists considered the duration of the group sessions too short to personally motivate all employees and to properly explain how to install and use the Activator activity monitor. The recurrent nature of the group sessions and onsite meetings was considered as a new booster to use intervention components.

*“I: Did you feel like that group meeting stimulated the employees a little bit?*

*R: Yes, so afterwards you would see a temporary increase in the use of the equipment, because in the beginning the facilities were used occasionally, but after some time, they were barely used.”*—Manager 5.

However, some department managers expected even more support and a more proactive attitude during the onsite meetings from the occupational physiotherapists throughout the intervention.

*“I remember that the occupational physiotherapists sent an e-mail once and mentioned that we could call him if needed. But that’s quite a simple approach and there are a lot of people who think it’s not worth the effort, whereas if they [occupational physiotherapists] took on a more pro-active approach, I think people would contact you more easily or ask a question.”*—Manager 6.

## Discussion

This qualitative study aimed to identify barriers and facilitators from an organizational perspective that influenced the implementation of the Dynamic Work intervention at a Dutch insurance company and to explore differences in those barriers and facilitators between departments with a low, middle and high level of implementation. Implementation factors were identified on four levels, i.e. organization, department, implementers, and intervention. On the organizational level, implementation was facilitated by a healthy working culture and financial support, in which the company invests in the health of their employees. On the department level, implementation was mainly hindered due to lack of information at the start of the project, late delivery of Dynamic Work equipment, large group sizes, employee’s work load and work tasks, and the ongoing reorganization. Department managers’ role was considered as both enabling and hindering. On the implementer level, implementation was facilitated by familiarity with the company and alignment amongst implementers. On the intervention level, the non-obligatory nature of the intervention and the limited availability and technical problems of equipment hindered implementation. Especially the stimulating role of the department manager, late delivery of dynamic work equipment and group sizes varied between low and high implementing departments.

Existing research has shown that organizational readiness for change is an essential factor for successful implementation. Organizational readiness for change refers to “the extent to which organizational members are psychologically and behaviorally prepared to implement organizational change” [14]. Change management experts have stressed the importance of establishing organizational readiness for change before implementing change [15, 16]. In the organization where the intervention was implemented, a healthy work culture was established top down, led by a special team focused on healthy working. However, organizational readiness is important on all levels of the organization and some department managers might not have been ready, while others were not able to prioritize the intervention and provide sufficient leadership. Management support is an important facilitator for implementation of occupational health interventions [10], because group leaders can help a group achieve its goals and influence the beliefs and attitudes of group members [3, 17]. In the current study, managers of high implementing departments took more naturally the role as leader for change effort, stimulated their employees and acted as a role model. This implies that additional training for managers to become better leaders for change effort [18] and involvement of managers early in stages of intervention development and initialization is necessary to increase the effectiveness of future interventions [19].

The lack of differences found between varying degrees of implementation between departments might be explained by the fact that evaluation took place in only one organization and the way we classified departments into level of implementation. In the current study, the same intervention was delivered by the same implementers across departments of one company. Differences found between low and high implementers were mainly on the level of the department, such as delayed delivery, managers’ key role and job tasks. Differences might have been larger if this intervention was implemented and evaluated in another company, as is shown in previous research [20]. Furthermore, data used to calculate the implementation score was based on individual participant data, and then aggregated to department level [9]. However, we could not link all interview data to specific departments, for example occupational physiotherapists provided information about all departments they were involved in during one interview. This hindered extracting all details on department level. Future research could firstly identify low and high implementers, and then conduct interviews with a focus on identifying differences between low and high implementing departments.

### Strengths and limitations

A strength of the current study is that we explored perspectives of different stakeholders linked to both low and high implementing departments on perceived barriers and facilitators, which enhances the credibility of the results. The systematic approach for data analyses, including developing a detailed codebook and multiple iterations of analyses are also a strength of the study.

A limitation, however, is that transferability of the results to different settings is limited, because the intervention took place within a specific organizational context. Furthermore, we did not include data of employees participating in the intervention. Participant data hardly represented any reflection on organizational factors for implementation. Their views and experiences with regard to the intervention have been described in the process evaluation paper, in which quantitative and qualitative data were collected across the evaluation domains context (i.e. reach), implementation (i.e. recruitment and delivery), and mechanism of impact (i.e. experiences) [9].

## Conclusions

Various barriers and facilitators influenced the implementation of the Dynamic Work intervention, where the key role of department managers, late delivery of dynamic work equipment, and groups sizes varied between low and high implementing departments. These results can contribute to developing and improving implementation strategies in order to increase the effectiveness of future occupational health interventions.

## Supplementary Information

**Additional file 1.** Topic guide of semi-structured interview with occupational physiotherapist.

**Additional file 2.** Topic guide of semi-structured interview with Dynamic Work coordinator.

**Additional file 3.** Topic guide of semi-structured interview with department manager.

**Additional file 4.** Codebook for barriers and facilitators related to implementation of the Dynamic Work intervention.

## Data Availability

Data can be made available for non-commercial purposes upon request to the authors.

## Abbreviation

DW: Dynamic Work
