# Supplementary material for: Barriers and facilitators influencing the implementation of the occupational health intervention ‘Dynamic Work’: a qualitative study
Source: BMC Public Health. 2022 May 11;22:947. doi: 10.1186/s12889-022-13230-9 (PMC9097120; doi:10.1186/s12889-022-13230-9)
Supplement: Supplementary file 1 — Additional file 1. Topic guide of semi-structured interview with occupational physiotherapist. [file 12889_2022_13230_MOESM1_ESM.docx]

Additional file 1: Topic guide of semi-structured interview with occupational physiotherapist

| **Domain** | **Questions and Probe** |
| --- | --- |
| Warm up and demographic | Characteristics of the respondent:   - How old are you? - What is your background and education? - How many years of work experience do you have as occupational physiotherapist? - How many years have you been working at this insurance company? - How many years of experience do you have in delivering health promotion programs? |
| Participation | - What was the main reason you decided to become the coach for the Dynamic Work project? - How was this decision made? - What made you say ‘yes’? |
| Program delivery | - Which specific departments have you coached? - How did the delivery of the program go from your perspective? [prompt: differences between departments] - Did you have to arrange certain activities prior to: [prompt: facilities, materials, meetings]   - 1) Kick-off meeting, 2) Second Plenary Meeting, 3) On-site counselling meetings - What is your experience with the delivery of:   - 1) Face-to-face meeting with the head of the department; 2) the kick-off meeting; 3) the second plenary meeting?; 4) on-site counselling meetings? - Did employees approach you in between these meetings? What kind of questions were asked? - What is your overall experience with the delivery of the Dynamic Work program? [prompt: any differences between departments, why?] - If you could rate the overall program delivery of the Dynamic Work program for each department, what number would you give on a scale from 1-10?   - Prompt: What is the reason you did not give a lower number? Why not a 10?   - Were there situations or events within the departments that might have influenced the delivery of the program? I mean things such as personnel turnover, reorganizations etc. |
| Program aspects | - What is your opinion on the Dynamic Work materials? (Probe: Activator, Sit-stand desk, cycling workstation, Dynamic Work manual). - If you could rate the program aspects, what number would you give on a scale from 1-10?   - 1) Overall Dynamic Work project; 2) Sit-stand desks; 3) Cycling workstations;   4) Office balls; 5) Activator and app; 6) Manual   - Which parts of the program do you think were most valuable to participants? Why? - Which parts were not? Why? - Did you notice any unexpected or unanticipated consequences (positive or negative) for the participants, department or for yourself from delivering Dynamic Work? - Any parts of the Dynamic Work program that needed more support? - Did you skip certain meetings? Which ones? Why? - Did you add parts to the program [prompt: meetings, assignments, time, materials, et cetera]. What? Why? - Did you provide other physical activity opportunities outside of the program? Which ones? - Any suggestions on how the program can be improved? |
| Barriers and facilitators | - What helped you to deliver the Dynamic Work program? What contributed to its success? Why? Could you explain? - What got in the way or hindered your delivery of the Dynamic Work program? What was difficult? And why? Explain.   If not covered, prompt for:  - Was the team manager helpful? What did he/she do that helped/did not help?  - Did you have meetings with the other occupational physiotherapist?  - Were there practical issues during delivery of the Dynamic Work program?  - Were there any organizational issues?  - Were there any issues with the participants?  - Did you as a coach experience issues?  - To what extent is Dynamic Work compatible with your other work at the insurance company?  *Carefully repeat all barriers and facilitators cited by the respondent, ask if there are more* |
| Sustainability | - Would you like to continue delivering the Dynamic Work program at the end of the research project? Why? Explain. - Have you done anything so far to continue the Dynamic Work program? - What are barriers to continue delivering? - What would be your advice to occupational physiotherapists in case they would like to deliver the Dynamic Work program? What are the most important things they need to consider for a successful implementation? - How can the program be funded in the future? What are your ideas? - Would you recommend the Dynamic Work program to other companies / departments? Why? - Do you have any final remarks or suggestions for improvements of the Dynamic Work project? |
