# Supplementary material for: Barriers and facilitators influencing the implementation of the occupational health intervention ‘Dynamic Work’: a qualitative study
Source: BMC Public Health. 2022 May 11;22:947. doi: 10.1186/s12889-022-13230-9 (PMC9097120; doi:10.1186/s12889-022-13230-9)
Supplement: Supplementary file 2 — Additional file 2. Topic guide of semi-structured interview with Dynamic Work coordinator. [file 12889_2022_13230_MOESM2_ESM.docx]

## Additional file 2: Topic guide of semi-structured interview with Dynamic Work coordinator

| **Domain** | **Questions and Probe** |
| --- | --- |
| Warm up and demographic | Characteristics of the respondent:   - How old are you? - How many years have you been working at this insurance company? - What is your function at this insurance company? |
| Context | Dynamic Work is one of the projects within your ‘Healthy Working’ policy.   - What kind of projects is your department involved in? - How is the Dynamic Work project originally developed? - How were you initially involved in the Dynamic Work project? - What was your role during the Dynamic Work project? |
| Recruitment of departments | - What was your experience with recruitment of departments? - How many departments were initially approached for participation? - How were those departments selected? - What were reasons for departments to (not) participate? - Which recruitment strategies were used? What worked and what did not work? - What went well and what did not in motivating departments to participate? What would you do differently next time? - How was the decision to participate made? Who were involved and who made eventually the decision to participate? - Were there any differences of opinion regarding participation? And if yes, how were these solved? |
| Recruitment of occupational physiotherapists | - How were coaches recruited or assigned to deliver the Dynamic Work program? - How did this process go? Were there any difficulties? |
| Recruitment of participants | - What did you do to recruit participants? - Who were involved in the recruitment process? And what did they do? - Do you have any suggestions to improve recruitment of participants? |
| Program delivery | - What helped you with getting the Dynamic Work project up and running?   - What contributed to its success? Why. Explain   - What were barriers? What was difficult? What did not succeed? Why? Explain. - How did the delivery of the program go from your perspective? [prompt: facilitators and barriers] - What is your opinion about the Dynamic Work program?   - Do you have suggestions for improvement? - Were there situations or events within the departments that might have influenced the delivery of the program? I mean things such as personnel turnover, reorganizations etc.   *Carefully repeat all barriers and facilitators cited by the respondent, ask if there are more* |
| Sustainability | - Are you planning to continue delivering Dynamic Work at the end of the research project? Why? Explain. - Have you done anything so far to continue the Dynamic Work program? - What would help you to continue Dynamic Work? And which stakeholders do you need to involve? - What are barriers to continue delivering? - How can the program be funded in the future? What are your ideas and preferences? - Can you give a real estimate of how much it would cost to implement the Dynamic Work program at a new department (only program costs, no costs related to research activities)? - Would you recommend the Dynamic Work program to other companies? Why? - What would be the role of this insurance company in rollout? - What would be your advice to other companies in case they would like to deliver the Dynamic Work program? What are the most important things they need to consider for a successful implementation? |
