# Supplementary material for: Barriers and facilitators influencing the implementation of the occupational health intervention ‘Dynamic Work’: a qualitative study
Source: BMC Public Health. 2022 May 11;22:947. doi: 10.1186/s12889-022-13230-9 (PMC9097120; doi:10.1186/s12889-022-13230-9)
Supplement: Supplementary file 3 — Additional file 3. Topic guide of semi-structured interview with department manager. [file 12889_2022_13230_MOESM3_ESM.docx]

Additional file 3: Topic guide of semi-structured interview with department manager

| **Domain** | **Questions and Probe** |
| --- | --- |
| Warm up and demographic | Characteristics of the respondent:   - How old are you? - How many years have you been working at this insurance company? - What is your role within the insurance company (or during this project)? - How would you describe your department? [prompt: size, mean age, men-women, function]? - Did you participate in the Dynamic Work intervention? |
| Recruitment of departments | - How is your department recruited to participate in the Dynamic Work program? - What was the main reason you decided to participate? - Who made eventually the decision to participate? |
| Recruitment of participants | - VUmc approached your employees for participation in the Dynamic Work program. Did you take any additional actions upon this invitation? - What were reasons for your employees to NOT participate in the Dynamic Work program? - Any suggestions to improve the recruitment? |
| Program delivery | - How did the delivery of the program went from your perspective? I suggest we discuss each of the components of the Dynamic Work program. - [prompt] What contributed to its success? Why? Explain. - [prompt] What were barriers? What did not succeed? Why? Explain. - If you could rate the overall program delivery of the Dynamic Work program, what number would you give on a scale from 1-10? - [prompt] What is the reason you did not give a lower number? Why not a 10? - Any suggestions to improve program delivery? - Were there situations or events within your department or in the direct environment of your department that might have influenced the delivery of the program? I mean things such as personnel turnover, reorganizations etc. |
| Sustainability | - Are you planning to continue delivering the Dynamic Work program at the end of the research project? Why? Explain. - Have you done anything so far to continue the Dynamic Work program at your department? - What would help you to continue? And who would you involve? - What are barriers to continue delivering? - Would you recommend the Dynamic Work program to other companies/departments? Why? - What would be your advice to other companies/department in case they would like to deliver the Dynamic Work program? What are the most important things they need to consider for a successful implementation? |
