# Supplementary material for: Barriers and facilitators influencing the implementation of the occupational health intervention ‘Dynamic Work’: a qualitative study
Source: BMC Public Health. 2022 May 11;22:947. doi: 10.1186/s12889-022-13230-9 (PMC9097120; doi:10.1186/s12889-022-13230-9)
Supplement: Supplementary file 4 — Additional file 4. Codebook for barriers and facilitators related to implementation of the Dynamic Work intervention. [file 12889_2022_13230_MOESM4_ESM.docx]

Additional file 4: Codebook for barriers and facilitators related to implementation of the Dynamic Work intervention

| **Original domain name in TICD checklist** | **Domain** | **Code** | **Code in Atlas.ti** | **Description of the code** |
| --- | --- | --- | --- | --- |
|  | **0 Suggestions for improvement** | Suggestions for improvement | Suggestions for improvement | Suggestions mentioned for improvement of the program or program components. |
| **1 Guideline factors** | **1 Program factors** | **Factors related the intervention itself, materials, evidence for the program. Also includes the compatibility of the program with regular tasks in terms of how well the program fits the setting and the feasibility to run the program the way it is designed; also includes factors related to the development of the program (how it has been developed and the extent to which stakeholders were involved in this process).** | | |
|  | 1.1 Program factors | Length and intensity of program | Program-length and intensity F  Program-length and intensity B | Factors with regard to the length of the program, time in between program components and keeping the program alive among participants. |
|  | 1.2 Program factors | Participatory development | Program-development F  Program-development B | Factors with regard to the development of the program and the role of the occupational physiotherapists in development. |
|  | 1.3 Program factors | Available time and number of participants during meetings | Program-meeting duration and number of participants F  Program-meeting duration and number of participants B | Factors with regard to the duration of the meetings, available time to execute or adjust program content, number of participants in meetings, and (consequences of) absence and presence of employees during meetings. |
|  | 1.4 Program factors | Reaching participants during onsite meetings | Program-onsite meetings F  Program-onsite meetings B | Factors with regard to reaching participants during the onsite individual counseling meetings and the planning of these meetings. |
|  | 1.5 Program factors | Availability of desks placed | Program-desks F  Program-desks B | Factors with regard to the use, accessibility, availability and the number of desks placed, and employees having a fixed workplace. |
|  | 1.6 Program factors | Non-functioning of intervention components | Program-non-functioning F  Program-non-functioning B | Factors with regard to the non-functioning of intervention components (Activators, bikes) and solving these problems. |
|  | 1.7 Program factors | Inconveniences of intervention components | Program-inconvenience F  Program-inconvenience B | Factors with regard to (in)conveniences experienced while using the various intervention components. |
|  | 1.8 Program factors | Delivery of furniture components | Program-delivery furniture F  Program-delivery furniture B | Factors with regard to the delivery of the furniture and the collaboration with the facility company. |
|  | 1.9 Program factors | Added value of intervention components | Program-added value F  Program-added value B | Factors with regard to the added value of intervention components, whether they are used or not, or seen as contributing more/less to positive outcomes. |
| **2 Individual health professional factors** | **2 Occupational physiotherapist factors** | **Any factors that relate to the knowledge, skills, and qualities of occupational physiotherapists that are needed to deliver the program as designed; any factors that relate to the motivation, beliefs, attitudes of occupational physiotherapists that influence the implementation of the program.** | | |
|  | 2.1 Occupational physiotherapist factors | Perceived importance | Physio-importance F  Physio-importance B | Factors with regard to the opinion of the occupational physiotherapists about the importance of the intervention. |
|  | 2.2 Occupational physiotherapist factors | Knowledge before start of program (training) | Physio-knowledge start F  Physio-knowledge start B | Factors with regard to the knowledge the occupational physiotherapists received prior to the start of the program and appreciation for the training. |
|  | 2.3 Occupational physiotherapist factors | Perceived efficacy | Physio-efficacy F  Physio-efficacy B | Factors with regard to the opinion of the occupational physiotherapists about whether the intervention truly works. |
|  | 2.4 Occupational physiotherapist factors | Experience occupational physiotherapists | Physio-experience F  Physio-experience B | Factors with regard to the experience of the occupational physiotherapists and how this becomes visible in practice. |
|  | 2.5 Occupational physiotherapist factors | Compatibility work occupational physiotherapists | Physio-compatibility work F  Physio-compatibility work B | Factors with regard to whether delivery of the intervention fits in with regular tasks of the occupational physiotherapists. |
|  | 2.6 Occupational physiotherapist factors | Occupational physiotherapist delivery style | Physio-delivery style F  Physio-delivery style B | Factors with regard to the difference in background of the occupational physiotherapists and their personal styles in which they delivered the intervention. |
|  | 2.7 Occupational physiotherapist factors | Occupational physiotherapist knows company | Physio-knows company F  Physio-knows company B | Factors with regard to the extent in which the occupational physiotherapists were already familiar with the company and the departments. |
|  | 2.8 Occupational physiotherapist factors | Communication between occupational physiotherapists | Physio-communication F  Physio-communication B | Factors with regard to the communication between the occupational physiotherapists, how it went, how coordination took place, and the team feeling. |
| **3 Patient factors** | **3 Participant factors** | **Any factors that relate to the needs, preferences, or behaviour of participants regarding the intervention** | | |
|  | 3.1 Participant factors | Recruitment | Participant-recruitment F  Participant-recruitment B | Factors with regard to the recruitment of participants. |
|  | 3.2 Participant factors | Available time participants | Participant-time F  Participant-time B | Factors with regard to the available time of employees for participation. |
|  | 3.3 Participant factors | Position (job content) and workplace | Participant-function F  Participant-function B | Factors with regard to the position of employees, whether they have a fixed or flexible workplace, how many meetings they usually attend, differences between type of employees per department, differences between locations. |
|  | 3.4 Participant factors | Experience | Participant-experience F  Participant-experience B | Factors with regard to the positive or negative experiences of participants with the program and their opinion on various intervention components. |
|  | 3.5 Participant factors | Reach of intervention | Participant-reach F  Participant-reach B | Factors with regard to what type of people participate in the intervention, such as the fact that more enthusiastic types or the fact that people who do need the intervention are in fact harder to reach. |
|  | 3.6 Participant factors | Reason for participation | Participant-reason for joining F  Participant-reason for joining B | Factors with regard to reasons of people to participate. |
|  | 3.7 Participant factors | Commitment during intervention | Participant-commitment F  Participant-commitment B | Factors with regard to the commitment of participants during the intervention, such as certain behavioral patterns or their enthusiasm fading over time. |
| **4 Professional interactions** | **4 Factors related to professional interaction / networks** | **Any factors that relate to influences and wider local, national or international networks of department and physios, for instance: occupational physiotherapists influenced by professional organizations, professional networks, prevailing norms or opinions of colleagues. Also includes local collaborations with other partner organizations, such as healthcare organizations or universities.** | | |
|  | 4.1 Factors related to professional interaction / networks | Practical role research partner | Networks-Practical role research partner F  Networks-Practical role research partner B | Factors with regard to the practical role of the research partner during implementation, such as communication and solving problems. |
|  | 4.2 Factors related to professional interaction / networks | Professionalism research partner | Networks-Professionalism research partner F  Networks-Professionalism research partner B | Factors with regard to the professionalism of the research partner. |
| **5 Incentives and resources** | **5 Resources and incentives** | **Any factors that relate to financial resources (funding) and human resources (such as the availability of or access to physios) and any other resources needed to implement the program. Any factors related to financial and non-financial incentives, also includes factors related to quality monitoring as incentive** | | |
|  | 5.1 Resources and incentives | Available budget company | Resources-budget F  Resources-budget B | Factors with regard to the budget for the program, such as the costs for occupational physiotherapists and the flexibility of coaches. |
| **6 Capacity for organizational change** | **6 Capacity for organizational change** | **Any factors that relate to the mandate, decision making, leadership, organizational regulations, organizational rules or policies, the priority to make a change. Also includes department culture aspects (such as internal communication channels and factors that relate to “that is how we do things here in the department”)** | | |
|  | 6.1 Capacity for organizational change | Scheduling meetings | Organization-planning F  Organization-planning B | Factors with regard to the planning of the meetings, such as reaching team managers, support for planning and unforeseen circumstances that influence planning. |
|  | 6.2 Capacity for organizational change | Recruitment departments | Organization-recruitment department F  Organization-recruitment department B | Factors with regard to the recruitment of the department, such as recruitment message, reason for participation, who made the decision for a department to participate, effect on productivity, etc. |
|  | 6.3 Capacity for organizational change | Reorganization | Organization-reorganization F  Organization-reorganization B | Factors with regard to the reorganization, such as its influence on participation, presence of employees during meetings and work climate in the departments. |
|  | 6.4 Capacity for organizational change | Support colleagues | Organization-colleagues F  Organization-colleagues B | Factors with regard to the support and opinions of colleagues about the intervention and the usage of intervention components. |
|  | 6.5 Capacity for organizational change | Support and commitment managers | Organization-manager F  Organization-manager B | Factors with regard to the managers, such as a change of managers within departments; the commitment, participation, enthusiasm and priorities of managers; and whether managers are leading by example. |
|  | 6.6 Capacity for organizational change | Support and facilitation by DW coordinators | Organization-project coordinator F  Organization-project coordinator B | Factors with regard to the support by DW coordinators, their flexibility and their functioning as point of contact within the company. |
|  | 6.7 Capacity for organizational change | Support of direction | Organization-board F  Organization-board B | Factors with regard to the support of the direction, their authority and top-down decision to participate. |
|  | 6.8 Capacity for organizational change | Vision and standards of company | Organization-vision company F  Organization-vision company B | Factors with regard to the vision and standards held by the company about sitting. |
| **7 Social, political and legal factors** | **7 Social, geographical, political, legal factors** | **Factors that relate to the wider social and political environment, legislation, political decisions, macro budgets, corruption, influential stakeholders** | | |
|  | 7.1 Social, political, legal factors | Norm | Context-norm F  Context-norm B | Factors with regard to the norm and the general acceptation of the ‘sitting is the new smoking’ phenomenon. |
